# Targeted RNA next generation sequencing analysis of cervical smears can predict the presence of hrHPV-induced cervical lesions

**DOI:** 10.1186/s12916-022-02386-1

**Published:** 2022-06-09

**Authors:** Karolina M. Andralojc, Duaa Elmelik, Menno Rasing, Bernard Pater, Albert G. Siebers, Ruud Bekkers, Martijn A. Huynen, Johan Bulten, Diede Loopik, Willem J. G. Melchers, William P. J. Leenders

**Affiliations:** 1 Department of Biochemistry, Radboudumc, Radboud Institute of Molecular Life Sciences, Geert Grooteplein 26, Nijmegen, 6525 GA The Netherlands; 2 Department of Medical Microbiology, Radboudumc, PO Box 9101, Nijmegen, 6500 HB The Netherlands; 3 Predica Diagnostics, Toernooiveld 1, Nijmegen, 6525 ED The Netherlands; 4 Department of Pathology, Radboudumc, PO Box 9101, 6500 HB Nijmegen, The Netherlands; 5 PALGA, De Bouw 123, Houten, 3991 SZ The Netherlands; 6 Department of Obstetrics and Gynecology, Catharina Hospital Eindhoven, Michelangelolaan 2, Eindhoven, 5623 EJ The Netherlands; 7 GROW, School for Oncology and Reproductive Biology, Maastricht University, Maastricht, The Netherlands; 8 Center for Molecular and Biomolecular Informatics, Radboud Institute of Molecular Life Sciences, PO Box 9101, Nijmegen, 6500 HB The Netherlands; 9 Department of Gynecology and Obstetrics, Radboudumc, PO Box 9101, Nijmegen, 6500 HB The Netherlands

**Keywords:** Cervical intraepithelial neoplasia, High risk human papilloma virus, Machine learning, Screening, Targeted RNA sequencing

## Abstract

**Background:**

Because most cervical cancers are caused by high-risk human papillomaviruses (hrHPVs), cervical cancer prevention programs increasingly employ hrHPV testing as a primary test. The high sensitivity of HPV tests is accompanied by low specificity, resulting in high rates of overdiagnosis and overtreatment. Targeted circular probe-based RNA next generation sequencing (ciRNAseq) allows for the quantitative detection of RNAs of interest with high sequencing depth. Here, we examined the potential of ciRNAseq-testing on cervical scrapes to identify hrHPV-positive women at risk of having or developing high-grade cervical intraepithelial neoplasia (CIN).

**Methods:**

We performed ciRNAseq on 610 cervical scrapes from the Dutch cervical cancer screening program to detect gene expression from 15 hrHPV genotypes and from 429 human genes. Differentially expressed hrHPV- and host genes in scrapes from women with outcome “no CIN” or “CIN2+” were identified and a model was built to distinguish these groups.

**Results:**

Apart from increasing percentages of hrHPV oncogene expression from “no CIN” to high-grade cytology/histology, we identified genes involved in cell cycle regulation, tyrosine kinase signaling pathways, immune suppression, and DNA repair being expressed at significantly higher levels in scrapes with high-grade cytology and histology. Machine learning using random forest on all the expression data resulted in a model that detected ‘no CIN’ versus CIN2+ in an independent data set with sensitivity and specificity of respectively 85 ± 8% and 72 ± 13%.

**Conclusions:**

CiRNAseq on exfoliated cells in cervical scrapes measures hrHPV-(onco)gene expression and host gene expression in one single assay and in the process identifies HPV genotype. By combining these data and applying machine learning protocols, the risk of CIN can be calculated. Because ciRNAseq can be performed in high-throughput, making it cost-effective, it can be a promising screening technology to stratify women at risk of CIN2+. Further increasing specificity by model improvement in larger cohorts is warranted.

## Background

Annually, 570,000 new cases of cervical cancer (CC) are diagnosed worldwide, with 310,000 attributable deaths [1–3]. Over 99% of CCs are associated with sexually transmitted and highly infectious high risk papilloma viruses (hrHPV) [4]. Because hrHPVs are causally involved in cervical dysplasia and cancer, CC-screening programs are increasingly based on molecular screening of cervical smears for the presence of hrHPV, followed by triage with cytology [5, 6].

In 2019, 9.8% of women tested positive for hrHPV in the Dutch CC screening program [7]. Of these hrHPV-positive women, 69% had normal cytology and were invited for a follow-up smear and cytology 6 months later. The remaining 31% hrHPV-positive women with cytological outcome ASC-US (atypical squamous cells of undetermined significance) or low- or high-grade squamous intraepithelial lesion (LSIL or HSIL) were referred for colposcopy, often accompanied by a biopsy. Of this group, only 37% was diagnosed with moderate or severe cervical intraepithelial neoplasia (CIN2 or CIN3) [7]. Whereas CIN3 lesions can be removed by a loop electrosurgical excision procedure (LEEP), the decision to treat CIN2 depends on factors like age and child wish, as LEEP can have serious side effects during pregnancy [8, 9]. Furthermore, in the majority of cases of low and medium grade HPV-induced CIN lesions (CIN1 and CIN2), these are spontaneously cleared within a year, allowing watchful waiting. In summary, the high sensitivity but low specificity of hrHPV testing for detecting CIN2+ results in high rates of overdiagnosis and overtreatment with an associated risk on adverse events. There is an unmet need for better triage tests to reduce the number of unnecessary referrals.

Productive hrHPV infections require the maintenance of HPV genomic DNA in an episomal state and repression of the immune response, conditions that are supported by the expression of, among others, the early HPV-E2 gene [10, 11]. This state is mostly associated with low-grade CIN (CIN1). Such infections are often transient and clear spontaneously [12]. Persistent infection may result in the integration of the viral genome in host DNA, which is frequently observed in CC [13] and high-grade CIN lesions [14]. Such integration is often associated with loss of expression of functional E2 and constitutive expression of the hrHPV-E6/7 gene [15–17]. E2/E6 RNA ratios are therefore lower in cancer than in CIN lesions [18]. Transcription from the E6/7 gene produces a bicistronic messenger RNA, encoding the E6 and E7 oncoproteins that are responsible for degradation of cell cycle regulator proteins P53 and pRB, and for altering transcription in infected cells [11, 19, 20]. As a result of functional loss of P53 and RB, uncontrolled proliferation accompanied by lack of functional DNA repair occurs, two principal requirements to start the oncogenic process. HPV E6/7 RNA assays have therefore higher specificity to detect CIN2+ (median 46%) than HPV DNA assays (38%) [21, 22]. This can be biologically explained because HPV DNA assays cannot distinguish between dormant, productive and oncogenic HPV infections.

For at least some hrHPV genotypes, the E6/7 gene contains a pseudo-intron that can be spliced out, resulting in the E6*I splice product. The E7 open reading frame is more efficiently translated from E6*I than from E6/7 mRNA, and it has been suggested that E6*I expression is associated with progression to higher grade CIN [23, 24]. There is debate in the literature if measuring hrHPV E6*I mRNA improves specificity to detect CIN2+ [23, 24]. Currently, there are no commercial tests available that can measure hrHPVE6*I RNA.

We previously reported on the potential of targeted RNA next generation sequencing (ciRNAseq) in several cancer types [25–27]. We also analyzed benign and malignant gynecological tissues with the technique [28] and showed its potential to concomitantly determine hrHPV oncogene activity and host gene activity. We showed that low ratios of hrHPVE2:E6/7 expression may indicate integration of the viral genome in the host genome [9].

Here, we used CC cell lines and cervical scrapes to test if simultaneous profiling of HPV oncogenes and of host genes that are implicated in hrHPV-oncogenesis can identify hrHPV-positive women who are at risk of having or developing CIN2+.

## Methods

### Cell lines and clinical material

HeLa cells and CaSki cells (a gift from Dr. A. Kaufmann, Charite University hospital, Germany) were cultured under standard conditions, trypsinized and fixated in PreservCyt solution (LBC, ThinPrep, Hologic Corp, Marlborough, MA, USA). Women participating in the Dutch CC screening program were informed that their residual cervical smear material in PreservCyt could be used for anonymized research and had the opportunity to opt out. Only left-over material from women who did not opt out was selected and analyzed after pseudonymization. Cytological classification of hrHPV-positive smears was performed according to the Bethesda system [29]. Cytological results and histological outcomes during follow-up were obtained from the nationwide network and registry of histo- and cytopathology in the Netherlands (PALGA; Houten, the Netherlands).

**Fig. 1:**
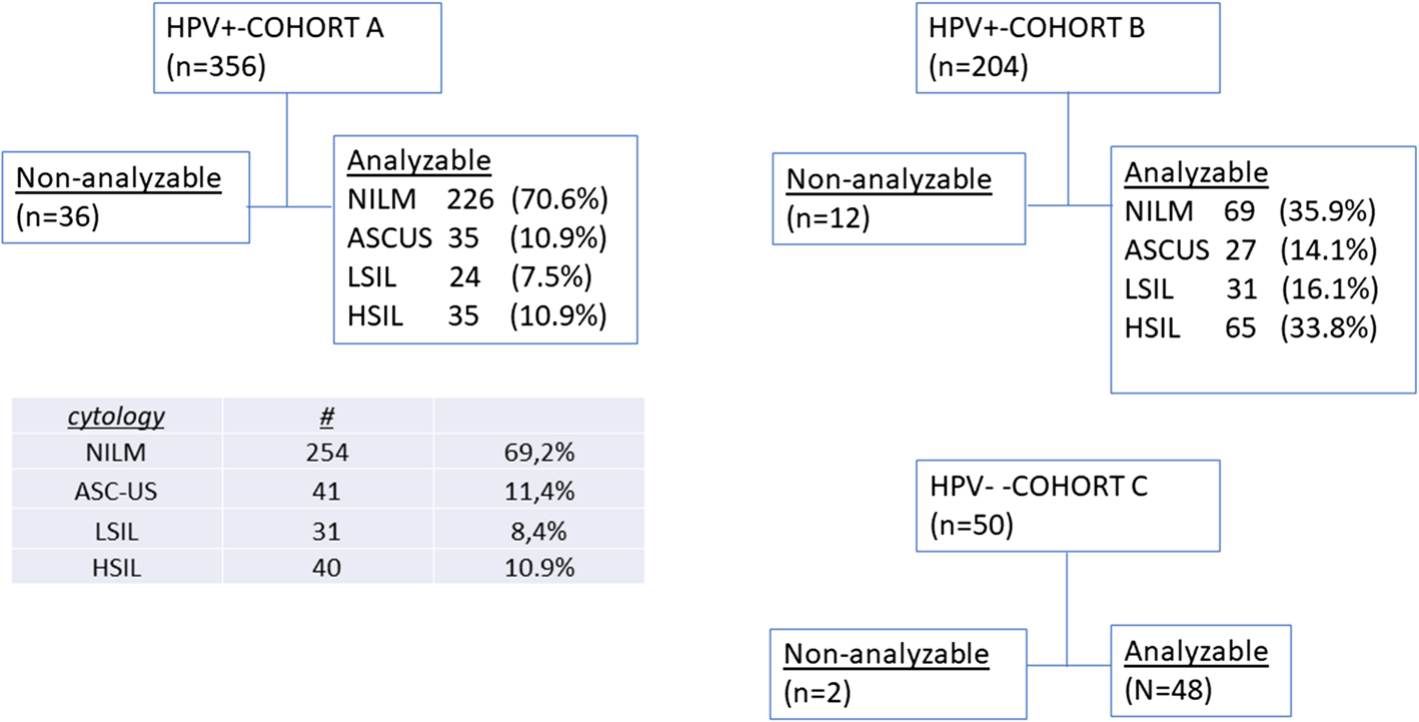
Summary of cohorts for analysis. Cohort A was collected at random from HPV-positive tested women, and cytology scores added afterwards; cohort B was selected for similarly sized groups with NILM, LSIL, and HSIL. Cohort C consists of random smears, tested negative for hrHPV-DNA. The table in Fig. 1 relates to cytological outcomes in cohort A

One cohort of hrHPV-DNA positive cervical smears (cohort A, *n* = 356) was randomly collected from the Dutch CC screening program. Another independent cohort (cohort B, *n* = 204) consisted of hrHPV-DNA-positive smears, selected for enrichment of specific cytological abnormalities. Furthermore, a cohort of 50 hrHPV-DNA negative scrapes (Cohort C) was analyzed. Cytology outcomes of cohorts A and B are summarized in Fig. 1. Clinical outcomes during follow-up of the combined cohorts A and B in relation to initial cytology scores are summarized in Table 1. For this study, women with cytology NILM (Negative for Intraepithelial Lesion and Malignancy) in both primary scrape and in return scrape during follow-up at 6 months were considered as “no CIN.” Median follow-up in this study was 7 months (range 6–709 days).

**Table 1:** Follow-up of all HPV-positive women (cohorts A and B)

1^st^ CYTO	NO FOLLOW UP	No CIN/ASCUS/LSIL/CIN1	CIN2+
NILM (*N*=321)	37 (11.5%)	264 (82.2%)	20 (6.2%)
ASCUS (*N*=65)	7 (10.8%)	47 (72.3%)	11 (16.9%)
LSIL (*N*=61)	6 (9.8%)	39 (63.9%)	16 (26.2%)
HSIL (*N*=109)	5 (4.6%)	22 (20.2%)	82 (75.2%)

### CiRNAseq

Five ml samples of residual scrape material in PreservCyt solution were centrifuged for 5 min at 2500×*g*. The pellet was lysed in 1 mL of Trizol reagent (Thermo Scientific), and RNA was isolated through standard procedures and dissolved in 20 μl nuclease-free water. Sixteen microliters with a maximum of 2 μg total RNA was treated with DNase, followed by cDNA generation using SuperscriptII (ThermoScientific) [28, 30]. The same protocol was applied to CaSki cells and HeLa cells after fixation in PreservCyt. To validate the efficacy of hrHPV specific probes, hrHPV cDNA amplicons were generated by RT-PCR using RNA from scrapes that were previously diagnosed as positive for RNA of different HPV genotypes. Amplicons were purified from agarose gel and equimolarly pooled. This pool was used as positive control in the assay.

The protocol for ciRNAseq was described before [28, 31]. In short, ~ 50 ng of cDNA or positive control was hybridized overnight with a set of 2394 single molecule molecular inversion probes (smMIPs), designed with MipGen software [32] to identify and quantitatively measure expression levels of a total of ~ 513 gene transcripts, including E2, E6/7, and E6* from hrHPV genotypes HPV 16, 18, 31, 33, 35, 39, 45, 51, 52, 56, 58, 59, 66, 68, and 73 and human transcripts encoding housekeeping proteins and enzymes involved in tyrosine kinase signaling, metabolism, DNA repair, oncogenes, tumor suppressor genes, and genes involved in immunity. Sequences were retrieved from hg38 (https://www.ncbi.nlm.nih.gov/assembly/GCF_000001405.26/) and from the PAVE database (https://pave.niaid.nih.gov/). The smMIP pool contained for each hrHPV genotype > 5 smMIPs that target non-overlapping, independent ROIs. During the capture reaction, extension arms in smMIP-cDNA hybrids were extended with KlenTaq polymerase, and smMIPs were circularized by Ampligase (both from Epicentre, Madison, WI). After enzymatic removal of non-reacted, linear smMIPs and cDNAs by exonuclease treatment, purified circular smMIPs were PCR-amplified with barcoded Illumina primer sets. PCR products of the expected length of 266 bp were purified with Ampurebeads (Beckmann Coulter Genomics, High Wycombe, UK) measured on Tapestation and subjected to Illumina Novaseq sequencing on an SP flow cell (2 × 150 bp reads).

### Data processing

Illumina output was barcode-decomplexed to produce forward and reverse FASTQ files for each sample. FASTQ files were processed by Seqnext software (JSI systems, Ettingen, Germany) to count the total number of reads, generated by each smMIP. To eliminate PCR amplification bias, all smMIPs were designed to contain an 8 N unique molecule identifier (UMI). Reads that contain the same UMI and have identical sequences were collapsed to unique counts, reflecting the number of individual smMIPs that were circularized in the assay. Seqnext settings allowed 2% mismatches, to prevent missing counts from intratypic HPV variants. To prevent false identification of low-risk (lr)HPVs with high sequence homology, ROI sequences with higher sequence homology to lrHPV were discarded by filtering. A sample was annotated as hrHPV-RNA positive if more than one hrHPV-specific read for E2, E6/7, and/or E6* was detected [28].

The total number of unique counts for each sample was used as a quality control for the efficiency of the capture reaction. Samples with an (arbitrary) total unique read count below 1000 did not match our quality requirements and were excluded from further analysis. Data were normalized by calculating

$$\frac{\mathrm{unique}\#\mathrm{reads}\ \mathrm{for}\ \mathrm{each}\ \mathrm{smMIP}\ }{\mathrm{total}\#\mathrm{unique}\ \mathrm{reads}\ \mathrm{for}\ \mathrm{all}\ \mathrm{smMIPs}}\ast 10\hat{\mkern6mu} 6$$

and expressed as FPM (fragment per million). The mean FPM value was calculated from all smMIPs reactive against that transcript. Mean FPM values were considered as gene expression value.

### Computational biology

To investigate if there is value in the data, we selected all HPV-positive tested women with a repeated cytology diagnosis NILM (considered as “safe”) and with an outcome CIN2+. Matrices of mean FPM levels of these samples were subjected to unsupervised agglomerative clustering using Manhattan distance and Ward.D2 method [25, 28]. Clusters were visualized using ClustVis [33]. Fisher’s exact test was performed to calculate significance of the asymmetric distribution of NILM and CIN2+ over the clusters. In a supervised analysis, differentially expressed genes between groups “safe” and “CIN2+” were identified by Mann Whitney U tests. Benjamini-Hochberg was used to calculate adjusted *P*-values, corrected for false discoveries. In parallel, mean FPM levels were log-transformed to achieve a normal distribution (after adding 0.1 to prevent log^0^), and differentially expressed genes were identified by a two-sided *T*-test. Genes that came out as differentially expressed by both tests with *P* < 0.05 and consistently over cohorts A and B were identified.

In the next step, decision tree models were built using the R package randomForest, version 4.6-14 [34] using ciRNAseq profiles of hrHPV-positive scrapes from women with outcome CIN2+ and women classified as safe (NILM in two consecutive scrapes). Data were randomly sampled into 5 training and validation sets (70/30) without allowing duplicates. For each pair, sets of 50 models were built. The models with the minimum number of false negatives were selected to form an aggregate set of five models. These models were then applied to ciRNAseq profiles from an independent external validation set of scrapes with known clinical outcome to calculate sensitivity and specificity.

## Results

### Gene expression profiling of PreServCyt fixed cell lines

We first investigated specificity and technical performance of ciRNAseq on HPV-positive cell lines CaSki and HeLa, and on a positive control sample, consisting of a mix of HPV cDNA amplicons. To mimic clinical scrapes as closely as possible, we fixated cultured cells in PreservCyt and stored aliquots of 10,000 cells for 1, 7, and 28 days as room temperature before proceeding to RNA isolation. Good quality data (as defined by total numbers of unique read counts) were obtained for samples even after 7 days of storage, whereas after 28 days, data quality was significantly less, though still interpretable (25-fold less unique read counts as compared to day 1, not shown). Table 2 shows a representative example of triplicate analysis of CaSki cells and HeLa cells, showing that in CaSki exclusively HPV16E2, E6* and E6/7 are detected, whereas in HeLa, HPV18E6/7 and E6* are detected, with relatively few reads from only a single HPV18E2-detecting smMIP. The lack of HPV18E2 reads in HELA cells was not a result of low-performance of the smMIPs because these performed well in the positive control (Tale IIB). Both cell lines were completely negative for all other hrHPV genotypes that are measured in this assay (data not shown).

**Table 2:** (A) Fragment of raw output with unique read counts of HPV16 and HPV18 E2, E67, and E6* with expression levels in CASKI and HELA cell lines. (B) Output of the same probes on a positive control sample containing hrHPV amplicons

A							B
***CELL LINE***		***CASKI***			***HELA***		amplicon controls
HPV16E2_smMIP1	237	197	286	0	0	0	868
HPV16E2_smMIP2	584	443	705	0	0	0	1533
HPV16E2_smMIP3	655	738	1056	0	0	0	1552
HPV16E2_smMIP4	37	23	45	0	0	0	81
HPV16E6*I smMIP5	871	842	1291	0	0	0	200
HPV16E6_smMIP6	636	576	905	0	0	0	1886
HPV16E6_smMIP7	25	19	41	0	0	0	926
HPV16E7_smMIP8	1654	1661	2369	0	0	0	1446
HPV18E2_smMIP1	0	0	0	57	44	45	426
HPV18E2_smMIP2	0	0	0	0	0	0	197
HPV18E2_smMIP3	0	0	0	0	0	0	2506
HPV18E6*I_smMIP4	0	0	0	11560	9834	9875	869
HPV18E6_smMIP5	0	0	0	989	882	815	543
HPV18E6_smMIP6	0	0	0	308	270	240	107
HPV18E7_smMIP7	0	0	0	13202	11229	11451	7548

### Profiling of cervical scrapes

Having established that PreservCyt fixation of CaSki and HeLa cells and storage at room temperature is compatible with ciRNAseq analysis, we proceeded with analysis of cervical scrapes that are routinely collected and stored in PreservCyt at room temperature. Storage time of samples was variable. We performed ciRNAseq analysis on 50 hrHPV-DNA negative scrapes and two independent cohorts of 356 and 204 hrHPV-DNA-positive scrapes from women, participating in the Dutch population-based screening program. Cytology characteristics of the randomly collected cohort A (Fig. 1) were in accordance with previously published national data [6], confirming that this cohort was representative for the hrHPV-positive Dutch population.

Quality of ciRNAseq data, expressed as total unique reads in a sample, varied from 0 to 1.12 million (mean 180,000, median 114,000). This high variability can be explained by differences in cellularity and RNA yield between samples. Dropout percentage (samples with less than 1000 total unique read counts) was ~ 8%, leaving 320, 192, and 48 analyzable samples in cohorts A, B, and C, respectively. HrHPV RNA was undetectable in all hrHPV-DNA negative scrapes that passed our quality control standards (Fig. 1 and data not shown).

### Associations of hrHPVE6/7 and HPV E6* gene expression with cytology

**Fig. 2:**
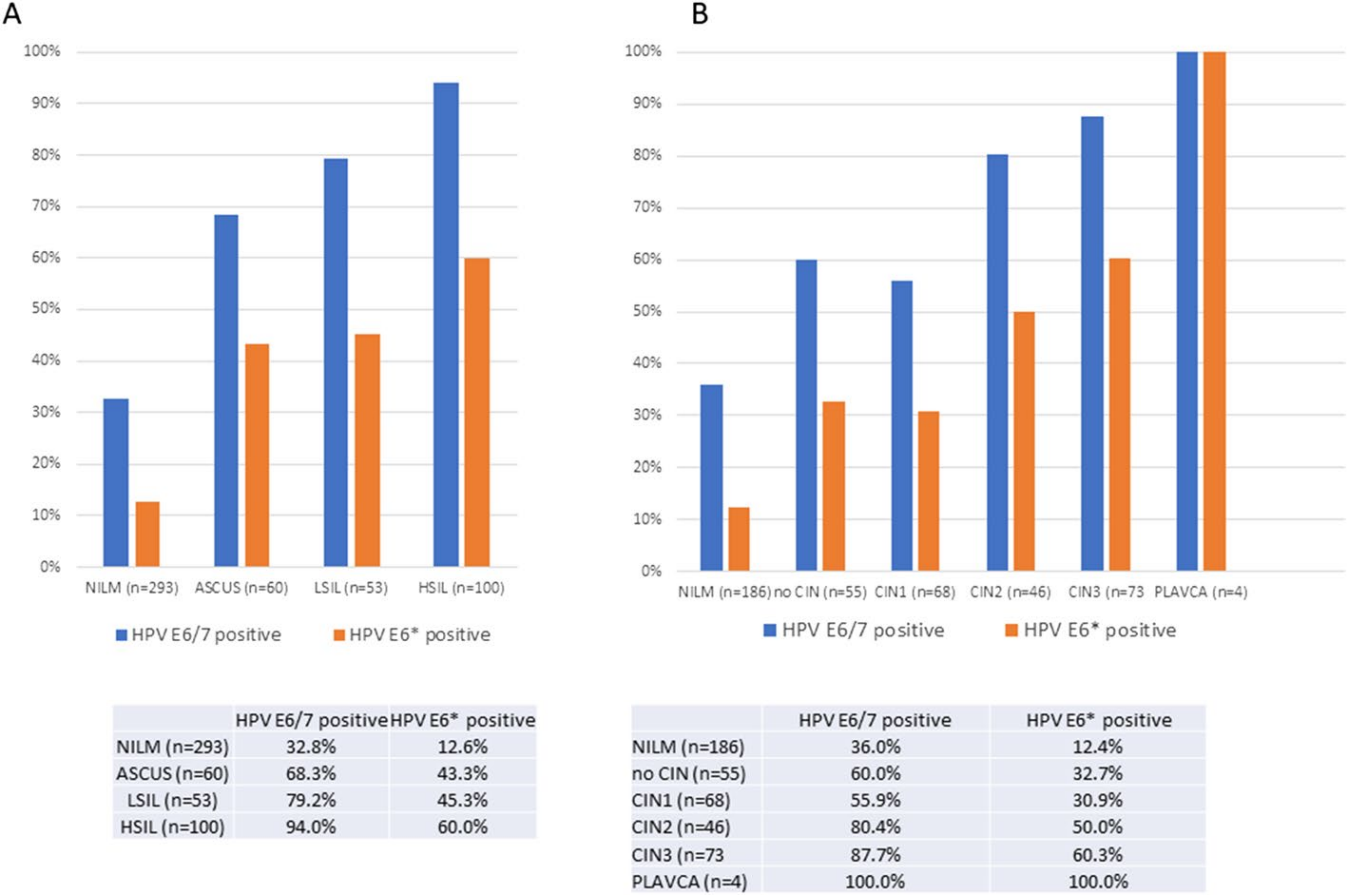
Positivity rates for hrHPVE6/7 and hrHPV E6*, related to cytology (**A**) and to colposcopy/histology outcome at follow-up (**B**)

We first investigated the randomly collected cohort A as a representation of hrHPV-positive women in the Dutch population. Results of hrHPV E6/7 RNA and E6* RNA expression in scrapes with cytology NILM, ASC-US, LSIL, and HSIL are summarized in Fig. 2A. In 33% of hrHPV-positive scrapes with the cytologic outcome NILM, hrHPV E6/7 RNA was detected. The percentage of hrHPV-E6/7 RNA positivity was 68%, 79%, and 94% in groups with cytology score ASC-US, LSIL, and HSIL, respectively. In 28/226 scrapes with no detectable hrHPVE6/7, we detected hrHPV-E2 mRNA without expression of E6/7 (not shown), suggestive of a productive state of the virus [16]. Of these women, 25 had outcome NILM, no CIN, or CIN1 (89%), 1 had an outcome CIN2 (4%), and 2 had outcome CIN3 during follow-up (8%).

HrHPV-E6* was expressed in 13% of all NILM-scrapes, 43% in ASC-US, 45% in LSIL, and 60% of HSIL. Similar patterns of hrHPV E6/7 and E6* expression were observed when hrHPV expression profiles in the first scrape were correlated to histology outcome with median follow-up of 7 months (see Fig. 2B).

### Associations of HrHPVE6/7 and HPV E6* gene expression with clinical outcome

In cohort B, similar frequencies of hrHPV RNA positivity were found as in cohort A (not shown). Therefore, cohorts A and B were combined in our further analyses. We first investigated to which extent the various hrHPV transcripts could predict the cytology diagnosis. Results are presented in Table 3 and show that hrHPV E6/7 RNA detection has the highest negative predictive value (85%), while hrHPVE6* RNA detection has the highest positive predictive value of detecting ASC-US or higher (75%, see Table 2 (B)). Also, for detecting outcome <CIN2, HPVE6/7 RNA had the better negative predictive value (85%) while hrHPVE6* RNA had the higher positive predictive value for detecting CIN2+ (59%, Table 3 (C)).

**Table 3:** A) distribution of HPV-RNA positivity over groups with cytology scores NILM and ASCUS+ (reason for referral to a gynecologist). B) distribution of HPV-RNA positivity in scrapes from women with an outcome <CIN2+ and >CIN2+ (median follow up 7 months)

**A**				**B**			
	NILM	ASCUS+	sens: 83%		<CIN2	CIN2+	sens: 81%
hrHPVE6/7 neg	197	36	spec: 65%	hrHPVE6/7 neg	217	37	spec: 48%
hrHPVE6/7 pos	96	177	NPV=85%	hrHPVE6/7 pos	165	155	NPV=85%
			PPV=66%				PPV=48%
			sens:52%				sens:56%
hrHPVE6* neg	256	103	spec: 75%	hrHPVE6* neg	318	85	spec: 59%
hrHPVE6* pos	37	110	NPV:71%	hrHPVE6* pos	75	107	NPV:79%
			PPV: 75%				PPV: 59%

### Associations of hrHPV genotypes with clinical outcome

**Fig. 3:**
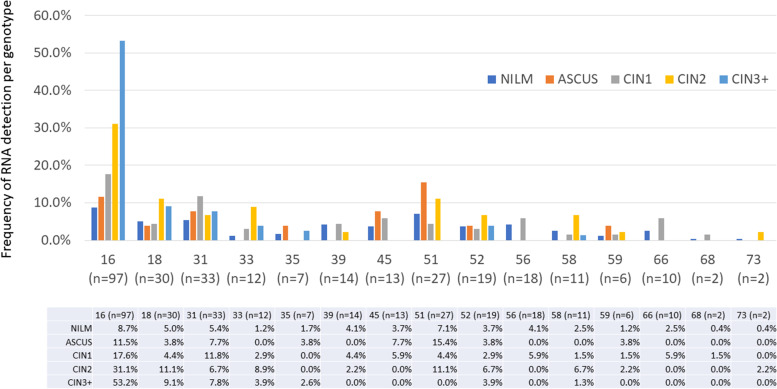
Distribution of HPV genotypes over groups of scrapes with different outcome (cytological or histological). Note that a number of HPV genotypes are exclusively found in lower grade lesions in this cohort

Sequence information from ciRNAseq can be directly used for hrHPV genotyping. Overall distribution of hrHPV genotypes in the two combined cohorts is presented in Fig. 3. As expected, HPV16 constitutes the majority of infections and was associated most with high-grade CIN. In this cohort, hrHPV genotypes 45/56/66/68 were underrepresented in CIN2+ relative to groups with no CIN/CIN1. In 72/298 hrHPV RNA positives (24%), multiple hrHPV genotypes were detected (not shown).

These data confirm that hrHPV-RNA detection has higher negative and positive predictive value than HPV-DNA testing but also show that hrHPV-RNA testing is not sufficient to stratify women who are at risk of CIN [35]. Therefore, additional biomarkers are needed.

### Additional value of host gene profiling

**Fig. 4:**
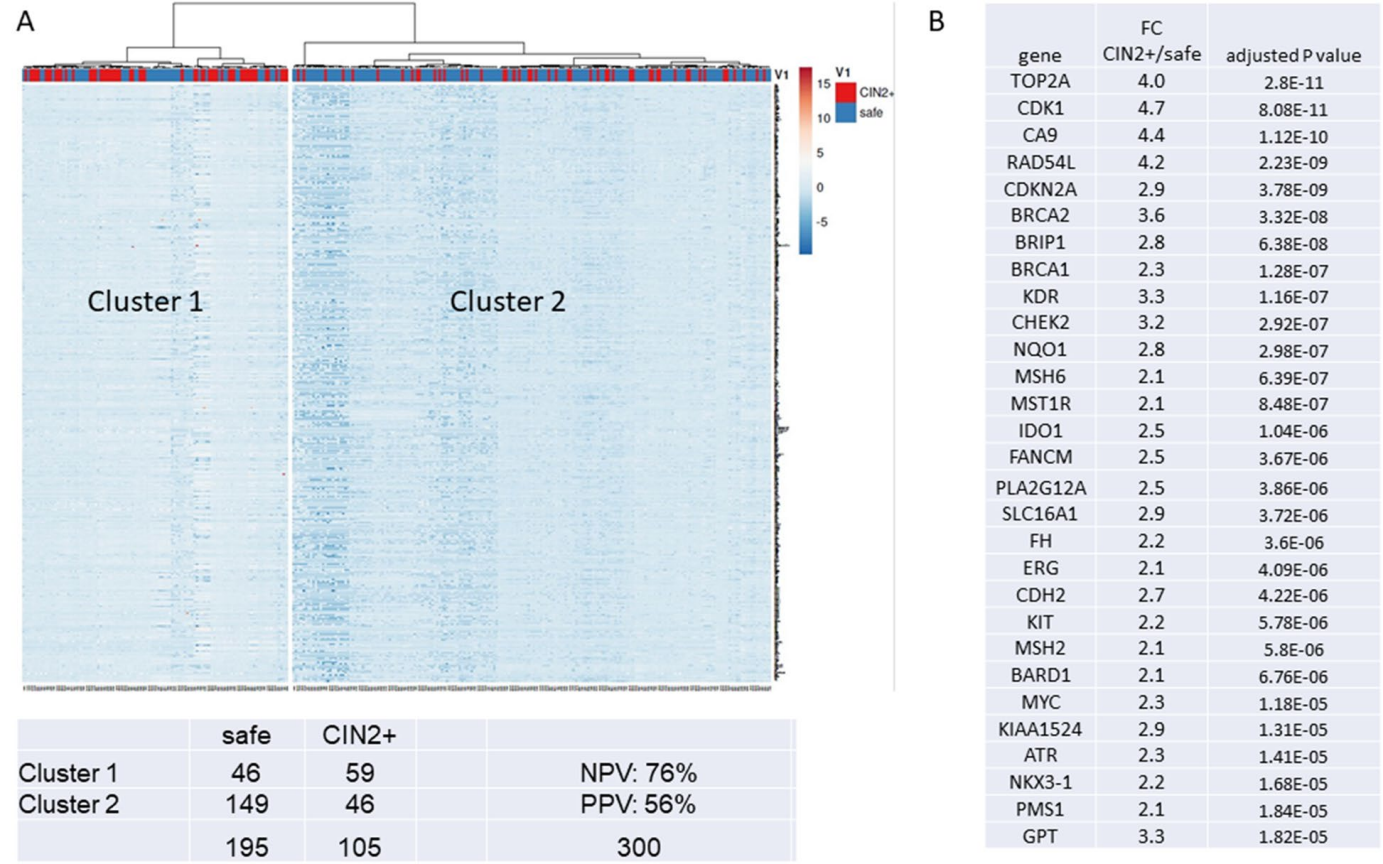
**A** Unsupervised hierarchal clustering of ciRNAseq data, excluding hrHPV transcript information, of groups defined as safe (NILM at primary and secondary cytology) and CIN2+. The table in **B** shows significantly overexpressed genes in smears of CIN2+ women, as determined by the Wilcoxon test (all highly significant with adjusted P-values < 0.00002)

In the process of hrHPV-induced oncogenesis, the transcriptional activity of the infected host cell changes [11, 19, 20]. To investigate if and how host gene expression levels correlate with histological outcome, we performed unsupervised hierarchal clustering of host gene expression data on all samples from women from with a cytology score NILM in first and second scrape, or with histology outcome no CIN, from here on referred to as “safe” (*n* = 195) and women with an outcome CIN2+ (*n* = 105) as described before [28]. We omitted scrapes from women with no follow-up and from women with an initial cytology ASC-US or higher, who had no CIN during follow-up, to prevent contamination of the group with underdiagnosed cases. Results in Fig. 4A show that the analysis yielded two main clusters. Distribution of CIN2+ and “safe” over the clusters was asymmetric with high significance (Fishers’ exact test, *P* < 0.0001). Even with this unsupervised clustering method, negative and positive predictive values of 76% and 56% were obtained for predicting CIN2+ from host gene expression data. Thus, these data show that host gene expression levels in scrapes can discriminate women who are safe from women with CIN2+.

We proceeded to identify the most prominent differentially expressed genes between groups “safe” and CIN2+ (Mann-Whitney *U* test, FDR< 0.00002, > 2-fold change in gene expression). This resulted in a set of 117 genes, a selection of which is shown in Fig. 4B. Relatively highly expressed genes in scrapes from women with outcome CIN2+ include genes involved in cell cycle regulation (e.g., *CDK1*, *CDKN2A*), DNA synthesis and repair (*ATR*, *BRCA1*, *BRCA2*, *BRIP1*, *FANCM*, *MSH2*, *MSH6*, *RAD54L*, *TOP2A*), kinase signaling (*KIT*, *NTRK1*, *PTPRZ*), metabolism (*CA9*, *GRIK5*, *NQO1*, *SLC16A1*), transcription factors (*MYC*), and immunity (*IDO1*).

**Fig. 5:**
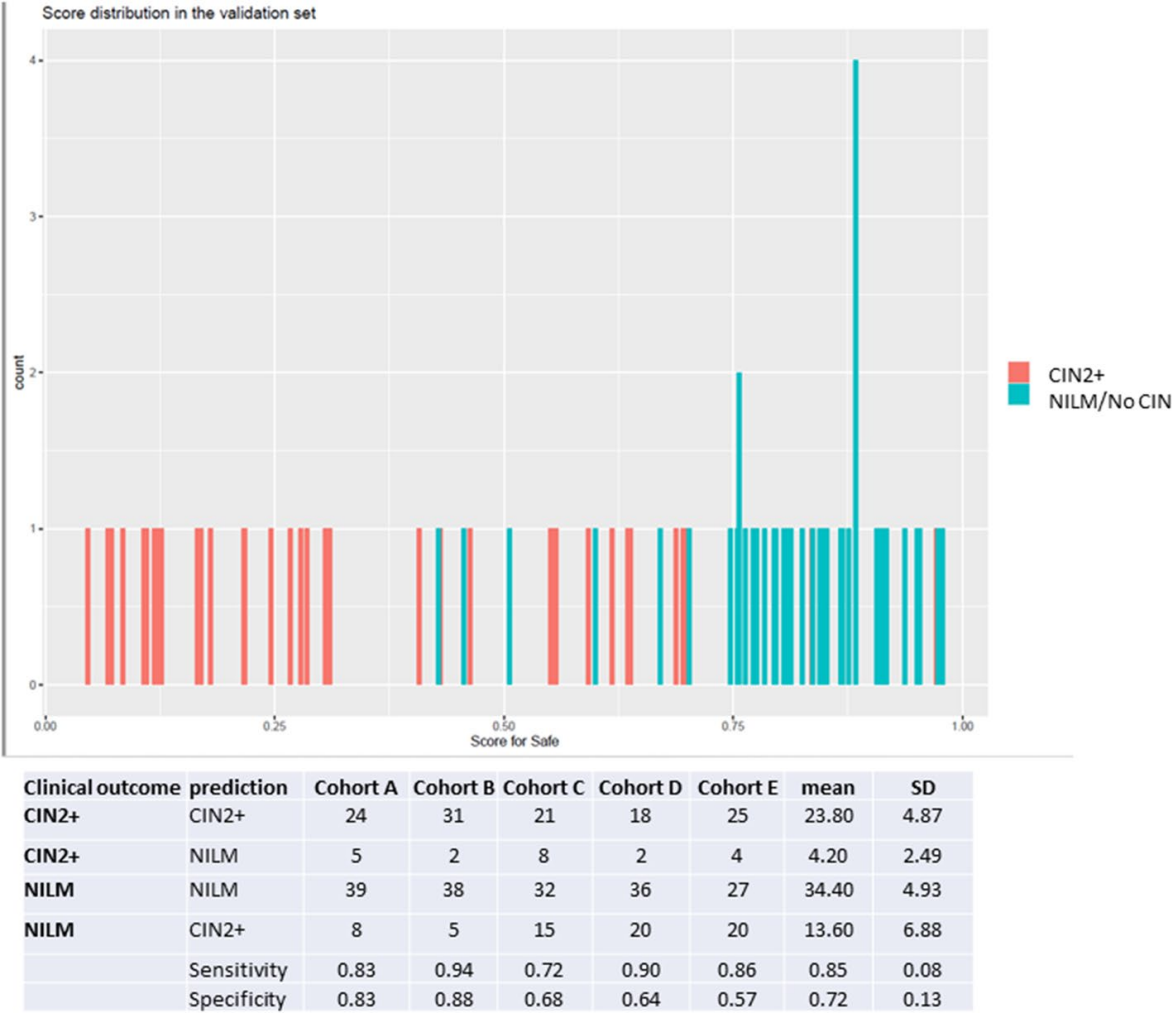
Outcome of the application of a random forest model, generated with ciRNAseq data from 360 smears, on an independent dataset of 63 smears. With a preset cutoff score of 0.8, all samples regarded safe (NILM at first scrape and repeat scrape) were correctly identified

To explore the potential of predicting CIN2+ based on the CiRNAseq data, we applied machine learning-based algorithms. Figure 5 shows one of the 5 independent cohorts that were analyzed by our random-forest based algorithm and shows that with a risk score cutoff of 0.7 (established during the building of the model) CIN2+ were identified by the model with a sensitivity of 85 ± 1% and a specificity of 72 ± 13%.

## Discussion

The introduction of primary hrHPV screening in prevention programs has led to increased numbers of referrals for colposcopy and biopsy of which more than 70% are in retrospect unnecessary. To reduce these numbers, there is an unmet need for reliable risk assessment tests [19, 36] that we addressed here using gene expression values measured with CiRNAseq.

CiRNAseq is a high-throughput technology of multiplexed targeted RNA sequencing that quantitatively measures RNAs of interest. Because smMIP probes can be selected to have exon-exon boundaries in their region of interest, the technique can be used for quantitative detection of splice variants of interest, such as E6*. In previous work, we demonstrated that in a subgroup of cervical cancers, HPV E6/7 RNA was found without expression of the E2 early gene [28]. To investigate this further and to confirm that PreservCyt fixation does not decrease RNA quality, we profiled PreservCyt-fixed cervical cancer cell lines CaSki and HeLa. For HeLa cells, only one of three E2-selective smMIPs recognizing the 5′-region of the E2 transcript was reactive against HeLa RNA. Since all E2-selective smMIPs were effective in control samples (Table 2 (B)), this confirms that the E2 gene is disrupted in this cell line [37], consistent with integration of the HPV gene in the HeLa genome with a break point in E2 [38]. Whereas E6/7:E2 ratios are elevated in cancers compared to CIN [18], it remains to be investigated if these ratios are reflected in cervical scrapes from women with high-grade CIN and cancer. This requires thorough investigation because expression of the E2 open reading frame on the transcript level is not necessarily reflected in the protein level, such as observed in CASKI cells [39, 40].

Our analysis of 560 cervical scrapes shows that ciRNAseq analysis of hrHPV-positive cervical scrapes can identify women who are at risk of high-grade CIN, by combining data on hrHPV gene- and host gene expression data. In the process, the technique also identifies HPV genotype. Analysis of the data confirms that HPV types 35/39/56/59 and 66 are mostly associated with low grade lesions [41]. Additionally, we found that in this cohort, HPV45, HPV58, and HPV68 were less frequently detected in HSIL than in lower grade cytology. This finding needs confirmation in larger independent cohorts.

In most bicistronic hrHPV E6/7 RNAs, the start codon for the E7 open reading frame (ORF) is close to the stop codon of the E6 ORF, leading to inefficient translation initiation of the E7 ORF. Removal of the E6 intron leads to a shortened E6 product, placing the E7 start codon in a context allowing efficient translation initiation. It has been suggested that expression of E6* is associated with higher grade CIN [23, 24], but we could not confirm this in this study. Additional studies in larger cohorts are required to investigate if E6* of specific genotypes have added value in risk predictions.

In this study, we found that ~ 70% of women with hrHPV-DNA positive, but hrHPV-RNA negative scrapes were diagnosed as NILM, suggesting that these cases concern latent infections, contributing to a higher negative-predictive value of ciRNAseq. On the other hand, for 7.5% of scrapes in which we could not detect hrHPVE6/7 RNA in the first scrape, women were diagnosed as CIN2+ in follow-up. Without exception, these cases were diagnosed at least 7 months after the first HPV-DNA positive scrape. Whether these cases concern newly acquired infections, or activation of latent infections diagnosed in the first scrape, is not known and requires that diagnosis of CIN lesions is accompanied by an additional scrape analysis with ciRNAseq. In this context, it is important to note that, whereas cervical cancers are considered to be caused by hrHPV, in a subgroup of HPV-DNA-positive cervical cancers, no HPV transcripts could be detected [42]. Also, the cancer genome atlas (TCGA) includes several HPV-negative cervix carcinomas [15]. Studies have shown that gene expression profiles of these cancers are distinct from those of HPV-positive cervical cancers, providing evidence that HPV-negative cervical cancers comprise a separate entity [43]. These cancers will be missed in screening programs that use HPV screening as a primary test. Additional clinical studies are required to test whether RNA profiling of cervical scrapes can identify HPV-negative gynecological cancers and which host cell RNA biomarkers should be measured for this purpose [35].

We argued that early-stage transcriptional alterations in clinically significant hrHPV infections would be readily detectable with ciRNAseq, giving additional predictive and prognostic value to HPV gene expression profiles [19, 36]. This hypothesis was confirmed in unsupervised cluster analysis of ciRNAseq data and machine learning which separated “safe” women from women with CIN2+ during follow-up.

We identified a set of human genes that are significantly higher expressed in scrapes from women with follow-up diagnosis CIN2+ compared to women with normal histology. Our results seem in part to be related to hrHPV biology: expression of hrHPVE6/7 oncogenes results in degradation of cell cycle gatekeepers TP53 and RB, leading to accumulation of DNA damage in cells with active DNA replication [44]. This may explain the upregulation of DNA damage sensor and repair proteins in scrapes with high-grade cytology. A well-known consequence of hrHPV E6/7 expression is upregulation of the cell cycle inhibitor gene *CDKN2A*, the gene encoding the p16^INK4a^ protein. Under physiological conditions, *CDKN2A* expression is mutually exclusive with expression of cell proliferation markers. However, transforming hrHPV infection results in a lack of RB, evoking expression of the *CDKN2A* product P16^INK4A^. The lack of RB also leads to continuous cell cycling. The co-expression of *CDKN2A* with proliferation markers such as CDK1 and proliferating cell nuclear antigen (PCNA) in CIN2+ [45–51] is a unique feature of malignant HPV-biology and was recapitulated in our ciRNAseq data. Interestingly, we also identified elevated expression levels of actionable genes. One example is IDO1, a protein involved in immune suppression and a possible target for therapy [52].

To investigate the value of ciRNAseq as a triage test on hrHPV-positive tested scrapes, we built prediction models using the random forest method on ciRNAseq profiles from scrapes with outcome “safe” or CIN2+ and tested the models on an independent cohort of scrapes with extreme outcomes (safe and CIN2+). The sensitivity and specificity of the models to predict CIN2+ in this group was respectively 85 ± 8% and 72 ± 13%. To improve specificity, large prospective clinical studies with sufficiently sized groups per hrHPV genotype and per cytology and long clinical follow-up are needed. Because of the small group sizes of low-prevalent hrHPVs, for this study, we grouped all hrHPVs. Our study shows that certain hrHPV genotypes are restricted to low-grade dysplasia only. If this can be confirmed in large studies, this biological knowledge can be implemented in the models. The same is true for detection of the HPV-E6* splice variant of different HPV genotypes. Other options to improve specificity of detecting CIN2+ could be the additional profiling of genes involved in immunity and inflammation, simply by predicting immune-mediated HPV clearance. If specificity can be raised, the technique could replace cytology as a triage test and has the potential to reduce overtreatment of healthy women that now receive a false-positive cytology diagnosis. The application of ciRNAseq can be seen in several scenarios, with advantages and disadvantages. It could be used as a primary screening test, which, according to our data, would immediately lead to a 70% reduction of false positive results (DNA-positive but RNA negative). Because in this case information on latent HPV-infection would be missed, effective screening would probably require retesting after 2 years. In a more realistic scenario, the test can be performed on HPV-DNA positive scrapes, substituting PAP tests, or can be performed as an addition to the PAP test. More research is required to determine what the most efficient scenario is with respect to cost-benefit.

## Conclusions

We here show the potential of ciRNAseq on cervical scrapes to detect expression of HPV oncogene RNAs from high-risk HPV genotypes, concomitant with genotyping and detection of expression levels from human host genes that are associated with CIN2+. Apart from hrHPV oncogenes, we identify a set of genes that are upregulated in scrapes from women with high-grade lesions. The combined hrHPV gene expression- and host gene expression data can be used to build decision-tree based models for more specific classification of women who need treatment because of increased risk of CIN.

## Data Availability

Data will be made available on request.

## Abbreviations

CC: Cervical cancer
CIN: Cervical intraepithelial neoplasia
FPM: Fragment per million
hrHPV: High risk human papilloma virus
NILM: Negative for Intraepithelial Lesion and Malignancy
smMIP: Single molecule molecular inversion probe
